# Surface Recovery Investigation of Silicone Rubber Composites for Outdoor Electrical Insulation under Accelerated Temperature and Humidity

**DOI:** 10.3390/polym13183024

**Published:** 2021-09-07

**Authors:** M. Hassan Raza, Abraiz Khattak, Asghar Ali, Safi Ullah Butt, Bilal Iqbal, Abasin Ulasyar, Ahmad Aziz Alahmadi, Nasim Ullah, Adam Khan

**Affiliations:** 1 High Voltage Engineering Laboratory, U.S.-Pakistan Center for Advanced Studies in Energy, National University of Sciences and Technology (NUST), Sector H-12, Islamabad 44000, Pakistan; mhassanraza423@gmail.com (M.H.R.); safibutt541@gmail.com (S.U.B.); bilaliqbalmgt@gmail.com (B.I.); abasin@uspcase.nust.edu.pk (A.U.); 2 U.S.-Pakistan Center for Advanced Studies in Energy, Department of Energy System Engineering, National University of Sciences and Technology (NUST), Sector H-12, Islamabad 44000, Pakistan; asghar@uspcase.nust.edu.pk; 3 Department of Electrical Engineering, College of Engineering, Taif University KSA, P.O. Box 11099, Taif 21944, Saudi Arabia; aziz@tu.edu.sa (A.A.A.); nasimullah@tu.edu.sa (N.U.); 4 Department of Electronics Engineering, University of Engineering and Technology (UET) Peshawar, Abbottabad 22010, Pakistan; adamkhan@uetpeshawar.edu.pk

**Keywords:** silicone rubber, silica, alumina trihydrate, SiO_2_, microcomposite, nanocomposite, hybrid, hydrothermal, polymer

## Abstract

Degradation of silicon rubber due to heat and humidity affect its performance in outdoor applications. To analyze the effects of high temperature and humidity on room temperature vulcanized (RTV) silicone rubber (SiR) and its composites, this study was performed. Five different sample compositions including neat silicone rubber (nSiR), microcomposites (15 wt% silica(SMC 15% SiO_2_) and 15 wt% ATH(SMC 15% ATH), nanocomposite (2.5 wt% silica(SNC 2.5% SiO_2_) and hybrid composite (10 wt% micro alumina trihydrate with 2 wt% nano silica(SMNC 10% ATH 2% SiO_2_) were prepared and subjected to 70 ˚C temperature and 80% relative humidity in an environmental chamber for 120 h. Contact angle, optical microscopy and Fourier transform infrared (FTIR) spectroscopy were employed to analyze the recovery properties before and after applying stresses. Different trends of degradation and recovery were observed for different concentrations of composites. Addition of fillers improved the overall performance of composites and SMC 15% ATH composite performed better than other composites. For high temperature and humidity, the ATH-based microcomposite was recommended over silica due to its superior thermal retardation properties of ATH. It has been proved that ATH filler is able to withstand high temperature and humidity.

## 1. Introduction

Silicone rubber (SiR) has been extensively studied for its application in the insulation industry. SiR is the most important polymer due to its unique behavior of recovery during aging [1]. The higher the temperature the faster will be the recovery; however, high humidity leads to more transfer time [2]. Silicone rubber has many useful applications such as high voltage insulators [3], plastic surgery [4] and biomedical engineering [5]. SiR is used for high voltage insulation because of its hydrophobic characteristics and hindrance to contamination that minimizes leakage current [6]. SiR has outstanding insulating properties as well as heat and weather resistance [7]. However, silicone rubber suffers from degradation due to environmental stresses such as humidity, salt fog, pollution, UV radiations, temperature, and acid rain, etc. Among these stresses heat and water the most dangerous factors. These environmental stresses deteriorate the inherent properties of polymeric insulators by inducing surface and structural changes [8,9]. The properties of silicone rubber can be further enhanced by using micro and nanocomposites [10,11]. The micro and nanocomposites still need to be tested under different accelerated stresses for the replacement of silicone rubber.

Several efficacious studies were conducted on the unique recovery behavior of SiR. For example, Faiza et al. [12] conducted hydrothermal aging of SiR/silica micro and nanocomposites for 1000 h. The silicone rubber nanocomposite exhibited better performance in terms of resistance to degradation and high recovery due to larger surface area and better chain intactness by nano silica. In s salt fog chamber, the hydrophobicity recovery of RTV SiR was studied under 15 kV applied voltage by Kim et al. [13]. Hydrophobicity loss occurred due to dry band arcing and the rate of hydrophobicity recovery was much faster in damaged surfaces than undamaged surfaces due to the transfer of low molecular weight (LMWs) components from material bulk to the surface. The main reason discovered by Hackam et al. [14] for the lack of affinity for water of SiR is due to the diffusion of LMWs components fluid from bulk to surface during the dry period.

Khattak et al. [10] investigated the hydrophobicity and life estimation of silicone rubber nanocomposites. Among them, SNC 5% silica performed better than nSiR and SNC 2.5% SiO_2_ in a leakage current test and STRI test. Mackevich et al. [15] concluded that each polymer material needs to be investigated individually due to the difference in their product design, formulation, and manufacturing conditions, and the user cannot predict a polymer behavior based on the study of another polymer. Liu et al. [2] discovered the hydrophobicity of silicone rubber depends on surface roughness, temperature, thermal radiation effects, UV spectrum, and contamination. In another study by Ali et al. [16], the hydrophobicity recovery of HTV silicone rubber after aging in saline solutions was examined. The contact angle increased at a faster rate during the evaporation of absorbed water in the initial hours. Tokoro et al. [17] studied the recovery of hydrophobicity of HTV SiR in saline solution as a function of time and temperature. It was reported that surface roughness increases with time, however, the contact angle remains the same. The hydrophobicity was decreased initially after immersion in solution and recovered in air, also high values of contact angle with increasing temperature were recorded.

In another study by Han et al. [18] it was discovered that tracking and erosion resistance greatly improved by adding ATH filler to HTV silicone rubber; however, loss in dielectric strength was recorded for higher loadings. In another study Cherney et al. [19] reported that silica and alumina trihydrate improved the overall thermal conductivity of composite material and provided resistance to erosion that normally occurs due to dry band arcing. Pradeep et al. [20] reported that the higher the loading of ATH, the better heat resistance for SiR composite. Recovery of hydrophobicity after heat stress is much better at high ATH levels compared to low concentrations of ATH. Due to high filler loading the thermal conductivity and heat dissipation are improved significantly up to certain limit.

In another study by Venkatesulu et al. [21] the erosion resistance of alumina-filled silicone rubber composites nanocomposites was analyzed. It was discovered that 4 wt% nanocomposites properties are comparable to 30 wt% microcomposite properties of ATH which is the basic function of filler distribution. Chang et al. [22] conducted a study on surface recovery of silicon rubber for high voltage outdoor insulation. They discovered the hydrophobicity recovery is affected by high temperatures. The higher the temperature, the faster will be the recovery. In another study by Charkraborty et al. [23] on HTV SiR under climate aging, it was concluded from the contact angle and wettability class that the thermally treated samples recovered hydrophobicity at a much faster rate. Meyer et al. [24] found out that the performance of polymer insulators under accelerated aging in a fog chamber, ATH filler (Al_2_O_3_ 3(H_2_O)) has better thermal conductivity than silica (SiO_2_).

Silicone rubber is well known due to its super recovery in comparison to other polymers, however its RTV-based composites have not been studied so far for this extraordinary property and compared for the best performance under high thermal and humidity stress. Most of the work has been done in the studies above for HTV silicone rubber and its composites. For the replacement of RTV silicone rubber with its composites, it is also important to test its composites under extreme stresses such as high temperature and water. After tests, the composites are analyzed using different techniques for recommendation of high voltage outdoor insulation. It is important to ascertain whether micro or nanocomposites are more desirable to use for high voltage outdoor insulation. Furthermore, it is also important which filler-based composite performs better when silica based, ATH based, or hybrid composite. The prime objective of this research is to find out the best composite for outdoor high voltage insulation according to the filler material type and size.

Keeping in view the aforementioned requirements, five samples of RTV SiR of different silica and ATH-based compositions were fabricated and subjected to the most important stresses of water and heat. They were analyzed and discussed in detail in this research to find the sample with the best recovery characteristics for the recommendation of outdoor high voltage insulation.

## 2. Research Approach

(1) Step 1:
  First the materials were developed with different filler concentration to make micro, nano and hybrid composites. There were five concentrations using silica to make micro, nano and hybrid composite. ATH filler was used to design microcomposite and hybrid.
(2) Steps 2:
  In the next step, samples were placed in an environmental chamber at 70 °C with 80% relative humidity. Stresses were removed and samples were subjected to testing every 30 h and after that step 2 was repeated until 120 h was completed.
(3) Step 3:
  The tests were performed after each cycle when the stresses of temperature and humidity were applied for contact angle measurement tests: Fourier transform infrared (FTIR) spectroscopy and optical microscopy to observe the hydrophobicity, functional group peaks and surface analysis.
(4) Step 4:
  In this step we collected and analyzed the results using Origin for FTIR graphic plotting, Dynamic 2500 for contact angle measurement and ScopeTek ScopePhoto software for optical microscopy analysis.

**Figure 1:**
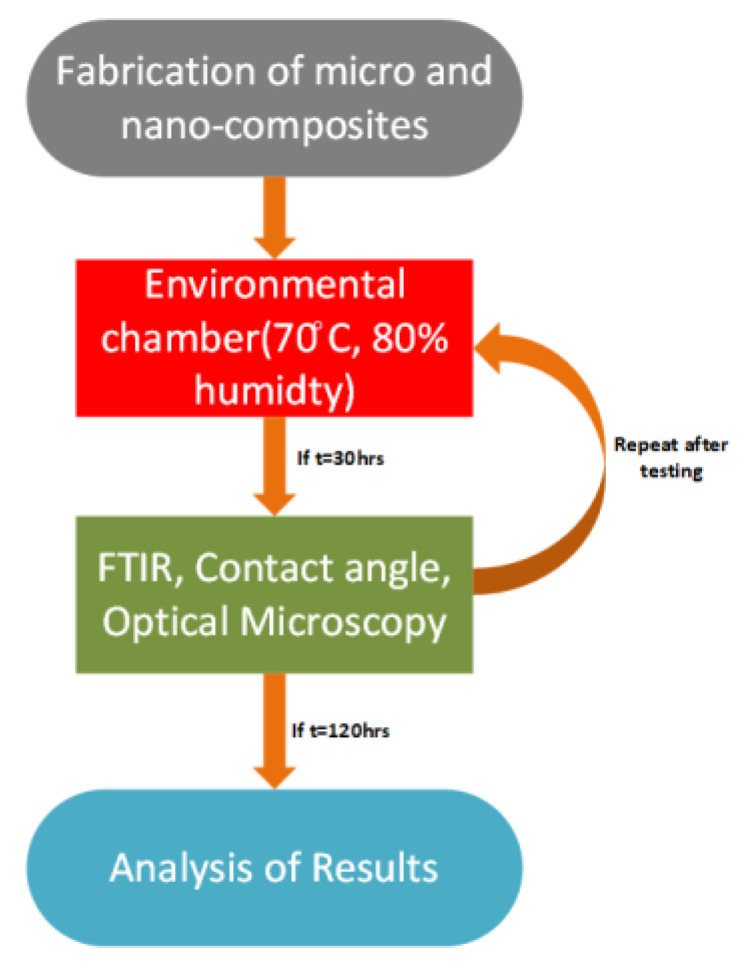
Flowchart of research approach.

Process of the research work is given in Figure 1.

## 3. Materials and Methods

### 3.1. Procurement of the Material

Room temperature vulcanized silicone rubber (RTV-615) was acquired by Lanxess AG chemicals, Leverkusen Germany. Nanosilica (12 nm) was received from Degussa, NJ, USA. Micro silica (5 µm) and micro ATH (5 µm) were received from Wuhan Newreach Chemicals in Wuhan, China, respectively.

### 3.2. Preparation of Sample

The samples were prepared with approximately 80 mm diameter and thickness ranging from 3–4 mm. The preparation of silicon rubber was done according to the percentage weight (% weight) of the filler and base polymer. For example, a hybrid (SMNC) composite containing 10 wt% ATH and 2 wt% nano silica contained 10 gm micro alumina trihydrate, 2 gm nano silica, and 88 gm of RTV 615 silicon rubber. The ratio that was maintained between curator and base polymer was 10:1. A sonicator was used for sample preparation using a shear mixer. Fillers were kept at 160 °C temperature for 16 h in a vacuum before preparation. In a Memert vacuum, SiR was placed at 460 mm Hg for a few hours. At a low speed of 3000 rpm, mixing was done in the dry filler of RTV 615-A to achieve proper wetting of filler. After that, at 5000 rpm mixing was performed until all the visible lumps disappeared in the mixture. To achieve maximum uniformity RTV 615-B was added in the mixture at low speed using the sonicator.

The prepared mixture was placed in a vacuum oven at 27 mm Hg for degassing and debubbling. The mixture was then poured into the mold to obtain the desired diameter and thickness and was cured for 24 h at room temperature. The samples were again heated for 4 h in the oven at 90 °C to remove any potential moisture. Table 1 consists of names, codes and loading of prepared samples.

## 4. Experimental Setup

The 5 samples were placed in programmable temperature and humidity chamber by UTSTESTER at 70 °C temperature and 80% humidity. The experiment was conducted for 120 h and after each 30 h interval, the samples were taken out of the chamber and measurement techniques were performed.

### 4.1. Measurement Techniques

#### 4.1.1. Contact Angle Measurement

The hydrophobicity of the samples was measured by static contact angle method using video contact angle (VCA) OPTIMA from ASTP. Distilled water was filled into a motorized syringe and volume of water droplet was maintained at 0.5 µL.

#### 4.1.2. Fourier Transform Infrared Spectroscopy

FTIR spectroscopy was used to assess the structural and chemical changes in absorbance form in the range of 4000 cm^−1^ to 650 cm^−1^. The mode used for FTIR was attenuated total reflectance (ATR). Agilent Technology’s Cary 630 was used for this purpose. 

#### 4.1.3. Optical Microscopy

Optical microscopy was employed to the study the surface of the samples at micro level using the lens magnification through visible light at a distance of 100 µm in reflectance mode. An Advanced Metallurgical Microscopy EQ-MM500T by MTI was used for this purpose.

## 5. Results and Discussion

### 5.1. Hydrophobicity Classification

**Figure 2:**
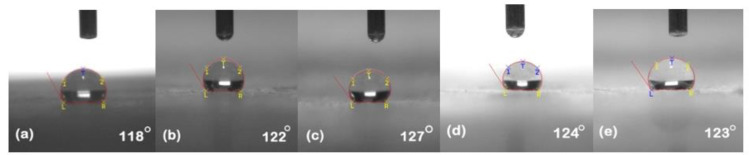
Contact angles of virgin samples (**a**) nSiR (**b**) SNC 2.5% SiO_2_ (**c**) SMC 15% SiO_2_ (**d**) SMC 15% ATH (**e**) SMNC 10% ATH 2% SiO_2_.

**Figure 3:**
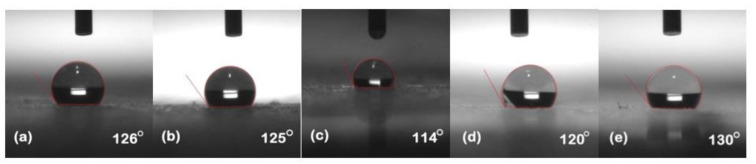
Contact angles of 30 h (**a**) nSiR (**b**) SNC 2.5% SiO_2_ (**c**) SMC 15% SiO_2_ (**d**) SMC 15% ATH (**e**) SMNC 10% ATH 2% SiO_2_.

**Figure 4:**
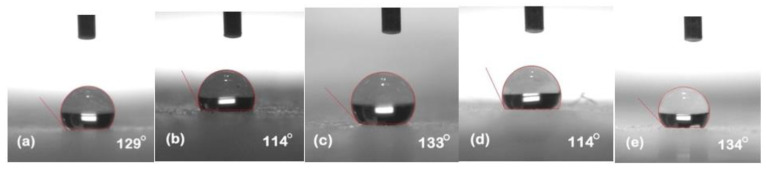
Contact angles of 60 h (**a**) nSiR (**b**) SNC 2.5% SiO_2_ (**c**) SMC 15% SiO_2_ (**d**) SMC 15% ATH (**e**) SMNC 10% ATH 2% SiO_2_.

**Figure 5:**
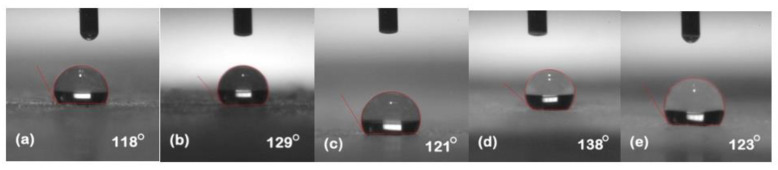
Contact angles of 90 h (**a**) nSiR (**b**) SNC 2.5% SiO_2_ (**c**) SMC 15% SiO_2_ (**d**) SMC 15% ATH (e) SMNC 10% ATH 2% SiO_2_.

**Figure 6:**
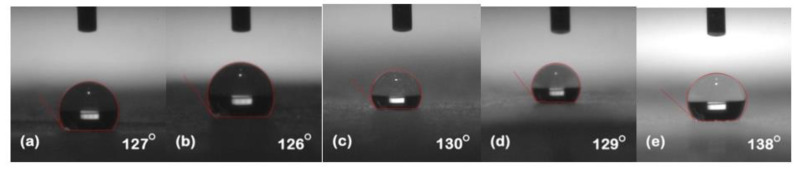
Contact angles of 120 h (**a**) nSiR (**b**) SNC 2.5% SiO_2_ (**c**) SMC 15% SiO_2_ (**d**) SMC 15% ATH (**e**) SMNC 10% ATH 2% SiO_2_.

The hydrophobicity of the samples was evaluated after each cycle using the static contact angle method. Silicone rubber and its composites exhibited their conventional behavior of high recovery hydrophobic behavior. Figure 2, Figure 3, Figure 4, Figure 5 and Figure 6 represent the contact angle of the virgin, 30 h aged, 60 h aged, 90 h aged, and 120 h aged samples, respectively.

All the samples expressed improvement in their hydrophobicity after stress was applied in the chamber. Neat SiR showed an improvement of 7.62% in its contact angle starting from 118° and ending on 127°. SNC 2.5% SiO_2_ expressed an improvement of 3.27% starting from 122° and ending at 126°. Similarly, SMC 15% SiO_2_, SMC 15% ATH and SMNC 10% ATH 2% SiO_2_ exhibited improvements of 2.36%, 4.03% and 12.19%, respectively. This improvement in hydrophobicity may have been due to the rotation of backbone chain and the transfer of low molecular weight components from material bulk to the surface of the silicone rubber [13,14]. It was also attributed to the abundance of free poly di-methyl siloxane (PDMS) chains that help silicone rubber to improve and retain its hydrophobicity. SMC 15% ATH expressed 11.29% improvement after the 4th cycle while SMNC 10%ATH 2% SiO_2_ expressed the most improvement of 12.19% after the 5th cycle.

Many authors have reported the cyclic behavior of silicone rubber [25,26]. The prime reason is due to the transfer of LMWs from bulk to the surface. The speed of recovery in silicone rubber is directly proportional to the amount of LMWs remaining in the material bulk [27]. Another reason for the recovery of hydrophobicity is due to the highly flexible siloxane chain and low molecular force between methyl groups [24,28]. The high energy of the bond of siloxane which holds out against the chain scission and Si-CH_3_ bond chain length is greater than the hydrocarbon methyl chain of other polymers. It has a highly mobile surface that allows the high free volumes of PDMS volume to move freely and reposition themselves.

The best contact angle of SMNC 10% ATH 2%SiO_2_ composite was due to the interaction between the silanol group at the surface of nano-silica and the SiR network due to the main group of SiR remaining intact against stresses. The ATH microcomposite had greater thermal conductivity compared to other composites. The formation of the exterior silica-siloxane protective layer was the reason for better hydrophobicity performance of silica with ATH as a thermal retardant as compared to nSiR. Silica nanocomposites were recommended for development of super hydrophobic behavior of SiR/SiO_2_ surface [29] and ATH’s thermal retardant properties, which might be the reason for SMNC 10% ATH 2% SiO_2′_s having the best hydrophobicity.

Overall the results showed recovery due to high temperature that speeds up recovery [30].

The summary of contact angles of all the samples is given in Table 2.

### 5.2. Optical Microscopy

**Figure 7:**
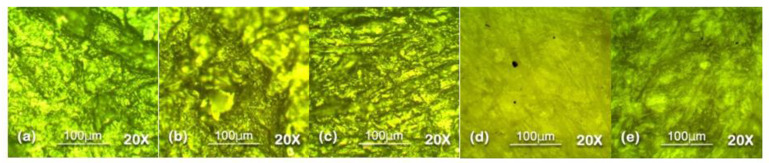
Unaged 20× Reflec (**a**) nSiR (**b**) SNC 2.5% SiO_2_ (**c**) SMC 15% SiO_2_ (**d**) SMC 15% ATH (**e**) SMNC 10% ATH 2% SiO_2_.

**Figure 8:**
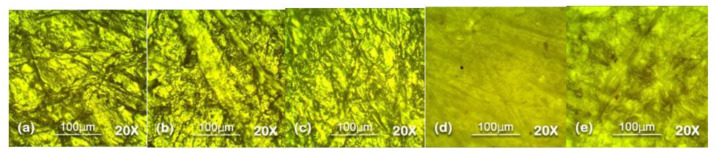
The 30 h 20× Reflec (**a**) nSiR (**b**) SNC 2.5% SiO_2_ (**c**) SMC 15% SiO_2_ (**d**) SMC 15% ATH (**e**) SMNC 10% ATH 2% SiO_2_.

**Figure 9:**
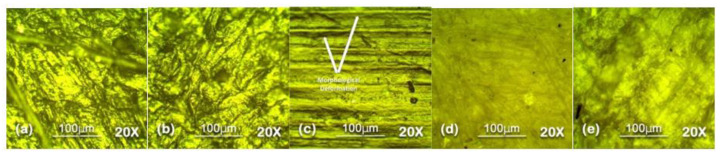
The 60 h 20× Reflec (**a**) nSiR (**b**) SNC 2.5% SiO_2_ (**c**) SMC 15% SiO_2_ (**d**) SMC 15% ATH (**e**) SMNC 10% ATH 2% SiO_2_.

**Figure 10:**
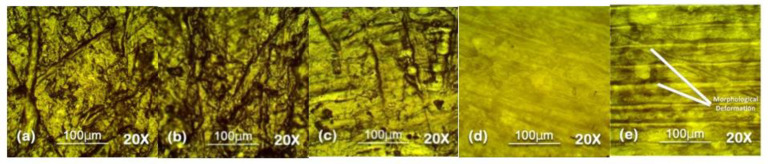
The 90 h 20× Reflec (**a**) nSiR (**b**) SNC 2.5% SiO_2_ (**c**) SMC 15% SiO_2_ (**d**) SMC 15% ATH (**e**) SMNC 10% ATH 2% SiO_2_.

**Figure 11:**
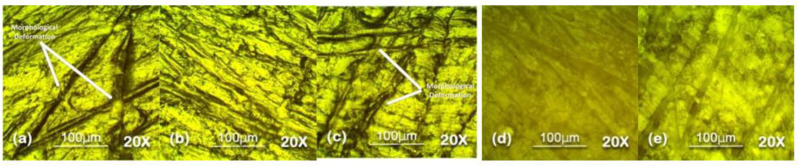
The 120 h 20× Reflec (**a**) nSiR (**b**) SNC 2.5% SiO_2_ (**c**) SMC 15% SiO_2_ (**d**) SMC 15% ATH (**e**) SMNC 10% ATH 2% SiO_2_.

Optical microscopy was used to analyze the surface topography at 100 µm level in reflectance mode. The results of optical microscopy are presented in Figure 7, Figure 8, Figure 9, Figure 10 and Figure 11. The surfaces of all the samples exhibited degradation due to high temperature and humidity stress. Random trails and pathways appeared on all the samples which caused unevenness and non-uniformity on the surface; however, the responses of all the samples were different from each other. Neat SiR expressed the most degradation due to the stresses applied. Neat silicone rubber exhibited loss of material that was the prime reason for the increase of roughness. It showed higher morphological deformation due to the heat. SMC 15% SiO_2_ also showed morphological changes on the surface but it was less than neat SiR. The surface topography of SNC 2.5% SiO_2_ was better than both neat SiR and SMC 15% SiO_2_ which was attributed to the greater surface intactness of the composite provided by nanosilica [22,23]. SMC 15% ATH and SMNC 10% ATH 2 SiO_2_ showed better behavior than all the samples. There were negligible cracks and ruptures in both the samples as compared to other samples. This was attributed to the thermal stability provided by the ATH filler. It is well documented that ATH filler provides better thermal conductivity and heat resistance on the surface of polymers. Among SMC 15% ATH and SMNC 10% ATH 2% SiO_2_, SMC 15% ATH performed better. The morphological deformation due to heat and water is significantly less in SMC 15% ATH as compared to SMNC 10% ATH 2% SiO_2_. The better surface behavior of SMC 15% ATH is due to the higher content of ATH filler as compared to SMNC 10% ATH 2% SiO_2_. This higher filler content of ATH provided even better thermal conductivity and resistance against thermal stress [20].

For nanocomposites, better surface morphology is because of filler-polymer bonding that stops the loss of material from the surface and causes speedy recovery. That explains the better behaviors of SNC 2.5 SiO_2_ compared to nSiR and SMC 15 SiO_2_ [29,30].

The summary of optical microscopy is given in Figure 7, Figure 8, Figure 9, Figure 10 and Figure 11 below.

### 5.3. Fourier Transform Infrared (FTIR) Spectroscopy

The FTIR spectroscopy technique was used to analyze the internal chemical changes of nSir, SNC 2.5% SiO_2_, SMC 15% SiO_2_, SMC 15% ATH and SMNC 10% ATH 2% SiO_2_. After each cycle, all the samples were analyzed in absorption form [31]. The important functional groups of silicone rubber and their respective wavenumbers are given in Table 3.

**Figure 12:**
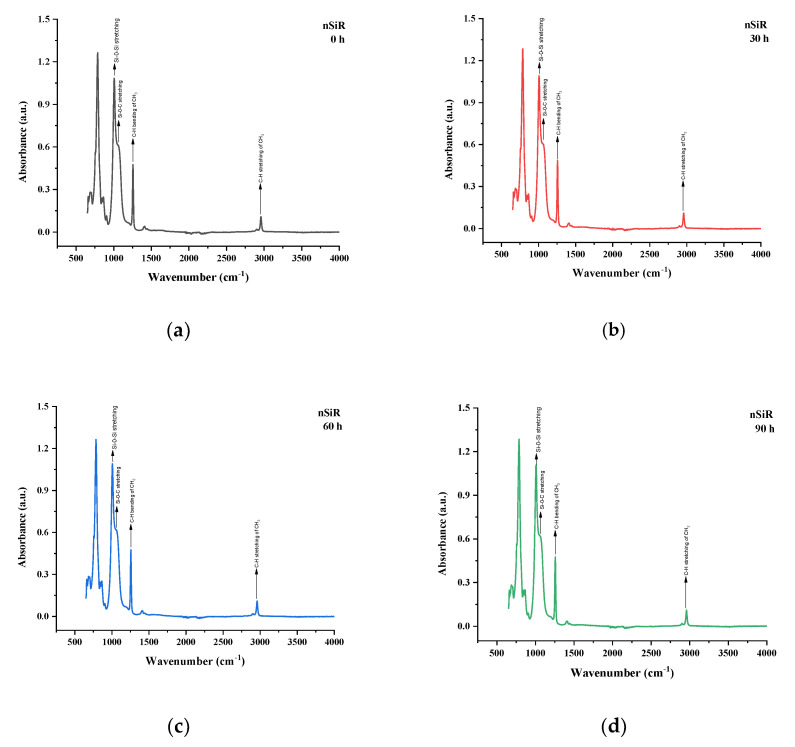

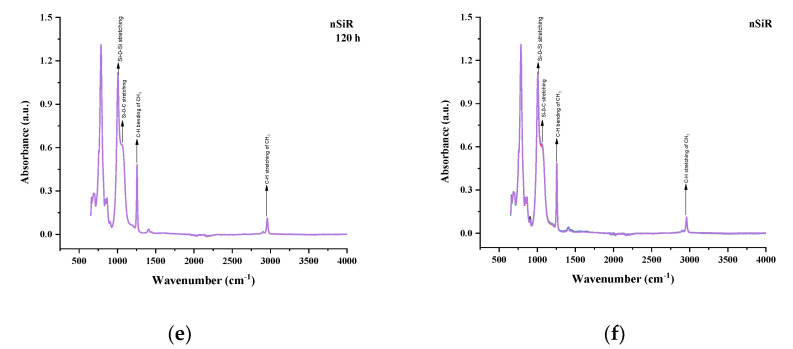
FTIR images of nSiR (**a**) virgin (**b**) 30 h (**c**) 60 h (**d**) 90 h (**e**) 120 h (**f**) combined.

**Figure 13:**
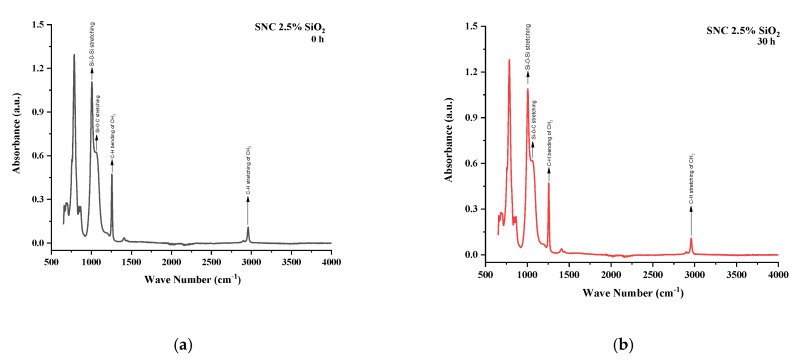

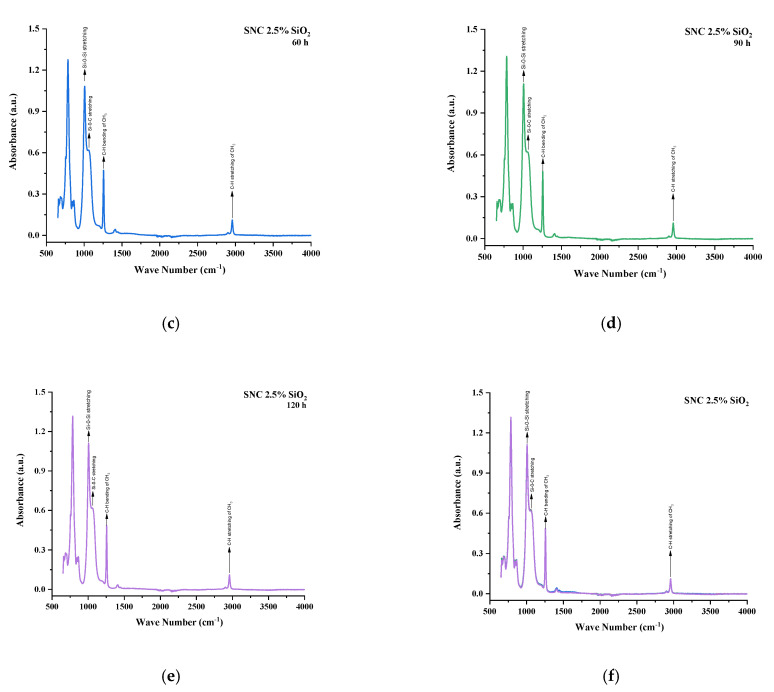
FTIR images of SNC 2.5% SiO2 (**a**) virgin (**b**) 30 h (**c**) 60 h (**d**) 90 h (**e**) 120 h (**f**) combined.

**Figure 14:**
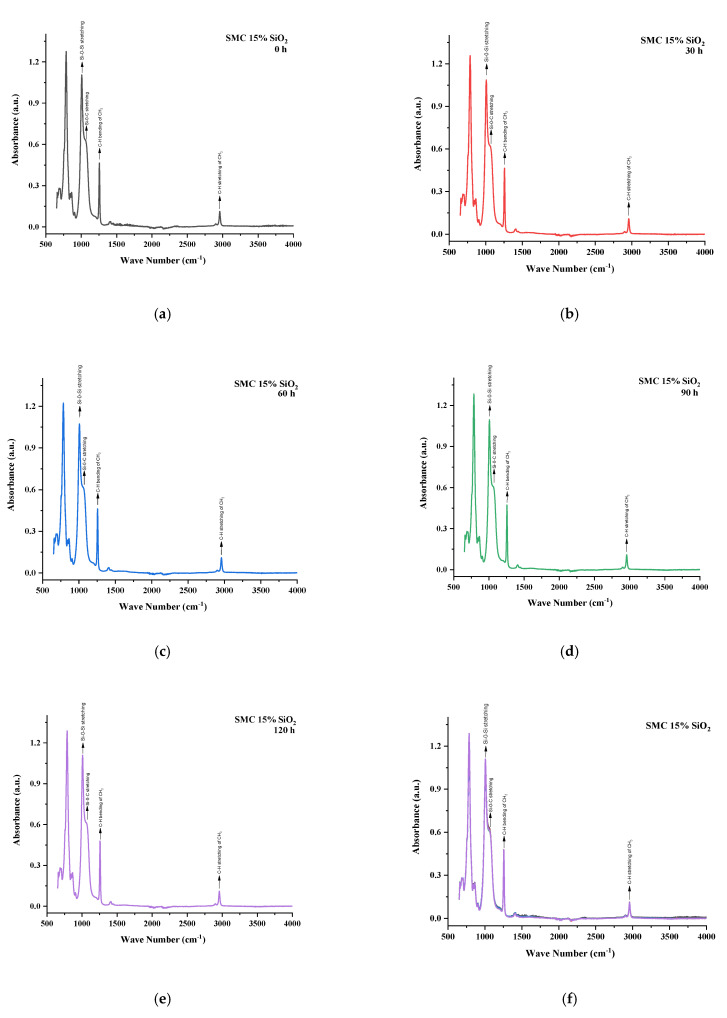
FTIR images of SMC 15% SiO_2_ (**a**) virgin (**b**) 30 h (**c**) 60 h (**d**) 90 h (**e**) 120 h (**f**) combined..

**Figure 15:**
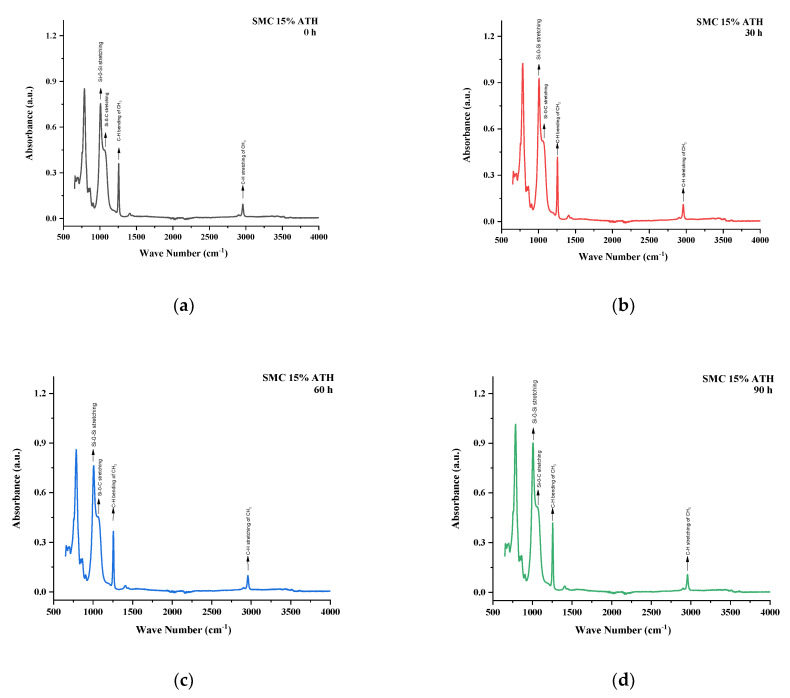

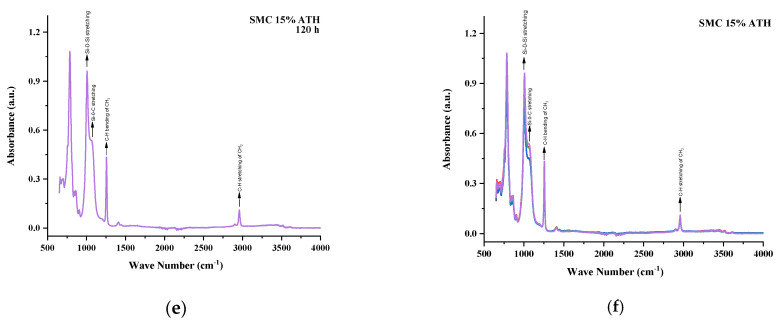
FTIR images of SMC 15% ATH (**a**) virgin (**b**) 30 h (**c**) 60 h (**d**) 90 h (**e**) 120 h (**f**) combined.

**Figure 16:**
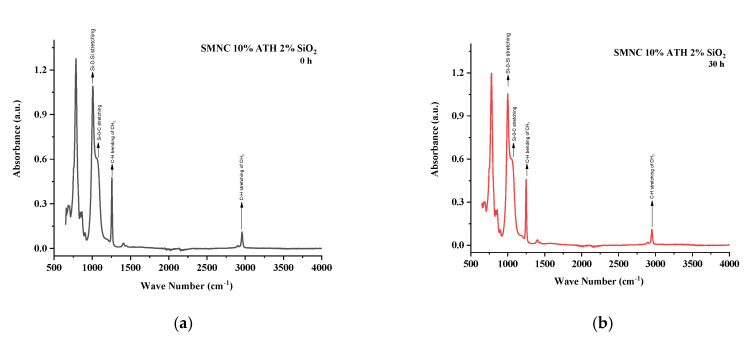

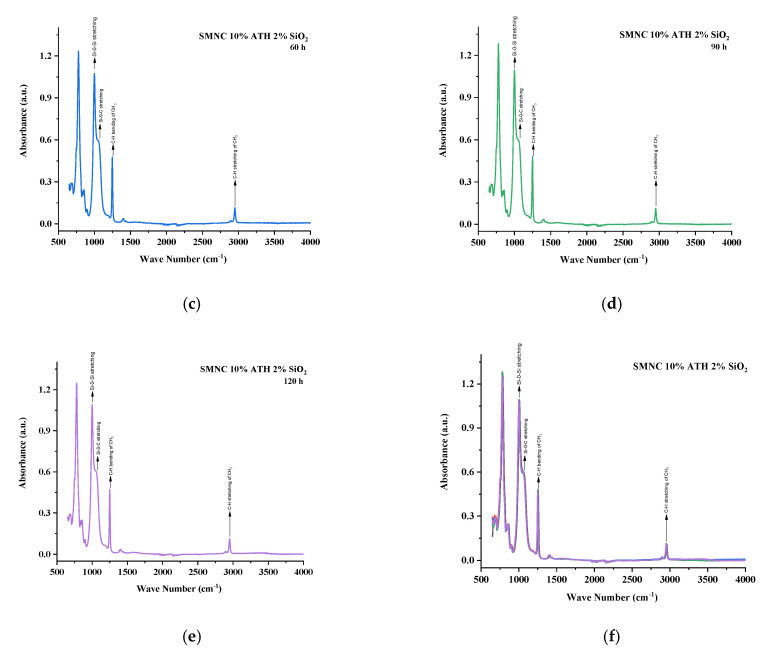
FTIR images of SMNC 10% ATH 2% SiO_2_ (**a**) virgin (**b**) 30 h (**c**) 60 h (**d**) 90 h (**e**) 120 h (**f**) combined.

All the samples expressed variations in absorption peaks which demonstrated continuous chemical change due to applied stresses. Spectrographs of all aged and unaged samples are present in Figure 12, Figure 13, Figure 14, Figure 15 and Figure 16.

In the nSiR there was an overall rise in peaks of functional groups. C–H stretching of CH_3_ at ~2963–2850 cm^−1^ showed absorption values of 102.7%, 101.7%, 102.8%, 103.7% of the virgin sample at 30 h, 60 h, 90 h and 120 h, respectively. Symmetric bending of Si–CH_3_ at ~1440–1400 expressed absorption values of 102.4%, 99.9%, 99.64%, and 102% of the virgin sample at 30 h, 60 h, 90 h, and 120 h, respectively. Similarly, other groups such as Si–O–C, Si–O–Si, and Si–(CH_3_)_3_ exhibited absorption values of 101.3%, 103.4%, and 102.15% after 120 h, respectively. The increase in hydrophobic methyl groups complimented the improvements in hydrophobic behavior of neat silicone rubber as seen in contact angle. In Table 4 the functional group values have been presented after each cycle for nSiR.

A similar trend of rising absorption peaks was seen in the case of SNC 2.5% SiO_2_. Symmetric C–H stretching of CH_3_ and symmetric CH_3_ bending expressed absorption values of 102.7% and 102.6% of virgin samples after 120 h, respectively. The absorption peaks of Si–O–C, Si–O–Si, and Si–(CH_3_)_3_ were 98.7%, 101.5%, and 100% after 120 h, respectively. In Table 5, the SNC 2.5% SiO_2_ FTIR absorbance values have been updated.

SMC 15% SiO_2_ also expressed both degradation and recovery in absorption peaks. Symmetric C–H stretching of CH_3_ and symmetric bending of CH_3_ showed absorption values of 96.5% after 30 h of aging but recovered to 98.3% of virgin sample after 120 h. Similarly, symmetric bending of CH_3_ showed recovery and degradation during aging and ended on 103.3% of virgin sample after 120 h. Other groups such as Si–O–C, Si–O–Si, and Si–(CH_3_)_3_ exhibited absorption values of 98.2%, 97%, and 100% of virgin samples after 120 h, respectively. The prime reason for the loss of peaks was due to filler–filler interactions in SMC 15% SiO_2_. This loss and recovery in absorption peaks was in harmony with the results of the contact angle for SMC 15% SiO_2_. In Table 6 the functional group values are provided for SMC 15% SiO_2_.

SMC 15% ATH expressed the most increase in absorption peaks. This behavior of SMC 15% ATH was also seen in the contact angle. The thermal retardant properties of ATH filler were responsible for the increase in contact angle as well as absorption peaks. Symmetric C–H stretching of CH_3_ and symmetric bending of CH_3_ expressed absorption peaks of 116% and 116% of virgin samples after 120 h. Other groups such as Si–O–C, Si–O–Si, and Si–(CH_3_)_3_ showed 120%, 123%, and 110% of virgin samples after 120 h. In Table 7 the functional group values have been provided for SMC 15% ATH.

In SMNC 10% ATH 2% SiO_2_, very minute variations in absorption peaks were observed. Symmetric C–H stretching of CH_3_ and symmetric bending of CH_3_ expressed absorption peaks of 100% and 98.8% of virgin samples after 120 h, respectively. Similarly, other groups such as Si–O–C, Si–O–Si, and Si–(CH_3_)_3_ showed 99.9%, 99.7%, and 100% of virgin samples after 120 h. In Table 8 the FTIR values have been presented for SMNC 10% ATH 2% SiO_2_.

The improved behavior of SMC 15% ATH than SMNC 10% ATH 2 SiO_2_ and silica-filled samples is due to thermal retardant properties of ATH and the higher filler content of ATH [20]. Also, it was reported that ATH is has more heat tolerance than silica [22] which explains why SMC 15% ATH has exhibited the best recovery as compared to other composites.

It can be seen from the analysis that FTIR peaks exhibited a gradual increase in absorption values for most of the samples that represent the transfer of hydrophobic methyl group from material bulk to the surface due to backbone chain rotation and the transfer of LMWs [13,14].

## 6. Conclusions

To investigate the chemical structure and surface analysis under accelerated temperature and humidity, five different composites of silicone rubber including neat silicone rubber(nSiR), two microcomposites (15 wt% silica(SMC 15% SiO_2_) and 15 wt% ATH(SMC 15% ATH), a nanocomposite (2.5 wt%. silica(SNC 2.5% SiO_2_) and a hybrid composite (10 wt% micro alumina trihydrate with 2 wt% nano silica(SMNC 10% ATH 2% SiO_2_) were prepared. They were subjected an environmental chamber under accelerated temperature and humidity stresses. Contact angle, FTIR and optical microscopy were recorded after 30 h and up to 120 h. Variations were observed in hydrophobicity, however an overall trend of recovery was documented. SMNC 10% ATH 2% SiO_2_ exhibited the highest contact angle of 138° due to the presence of nano silica along with ATH. In FTIR, recovery was also observed. SMC 15% ATH showed the most significant gain of 116%, 116%, 120%, 123% and 110% in symmetric C–H stretching of CH_3_, symmetric bending of CH_3_, Si–O–C, Si–O–Si, and Si–(CH_3_)_3_ functional group peaks respectively. SMC 15% SiO_2_ showed degradation in FTIR peaks due to filler–filler interaction. In optical microscopy, nSir and SMC 15% SiO_2_ showed the most morphological deformation due to thermally induced changes. SMC 15% ATH performed better and showed no cracks, holes or pathways. SMNC 10% ATH 2 SiO_2_ also had less changes in morphology due to the presence of ATH material, which has heat-resistance properties. It was quite clear from the analysis that SMC 15% ATH had greater recovery from the applied conditions due to the presence of ATH and its filler content as compared to other samples.

Based on the study and analysis above, SMC 15% ATH is recommended for outdoor high-voltage insulation purposes under severe conditions.

Only two stresses (water and heat) were applied for the analysis of sample compositions. Multiple stresses such as ultraviolet rays, acid rain and salt fog etc. can also be applied for better analysis of recovery behavior of silicone rubber and to understand the materials’ properties in a detailed manner.

## Figures and Tables

**Table 1:** Samples types and composition.

Sample Name	Filler Concentration (% wt.)	Sample Code
Neat SiR	0	nSiR
SiR Silica Nanocomposites	2.5%	SNC 2.5% SiO_2_
SiR Silica Microcomposite	15%	SMC 15% SiO_2_
SiR ATH Microcomposites	15%	SMC 15% ATH
SiR Hybrid composites	10%, 2%	SMNC 15% ATH 2 SiO_2_

**Table 2:** Comparison of contact angle of all composites after each cycle.

Time (h)	nSiR	SNC 2.5% SiO_2_	SMC 15% SiO_2_	SMC 15% ATH	SMNC 10% ATH 2% SiO_2_
0	118°	122°	127°	124°	123°
30	126°	125°	114°	120°	130°
60	129°	114°	133°	114°	134°
90	118°	129°	121°	138°	123°
120	127°	126°	130°	129°	138°

**Table 3:** Functional groups and their wave numbers (cm^−1^).

Functional Group	Wave Number (cm^−1^)
C–H stretching in CH_3_	~2963–2940
Si–CH_3_	~1280–1260
Si–0–C	~1110–1050
Si–0–Si	~1130–1000
Si–O stretching of O–Si(CH_3_)_3_	~875–865
Si–O stretching (Si–CH_3_)_2_	~850–790
Si–C stretching of Si–(CH_3_)_3_	~705

**Table 4:** Intensities of absorption peaks of nSiR from 0 to 120 h.

Functional Group	Wave Number (cm^−1^)	Absorbance				
		Virgin	30 h	60 h	90 h	120 h
C–H stretching in CH_3_	~2963–2940	0.107	0.109	0.109	0.110	0.111
Si–CH_3_	~1280–1260	0.473	0.485	0.473	0.472	0.483
Si–0–C	~1110–1050	0.612	0.612	0.620	0.622	0.620
Si–0–Si	~1130–1000	1.082	1.092	1.090	1.108	1.119
Si–O stretching of O–Si(CH_3_)_3_	~875–865	0.247	0.264	0.251	0.250	0.247
Si–O stretching (Si–CH_3_)_2_	~850–790	1.260	1.284	1.263	1.285	1.311
Si–C stretching of Si–(CH_3_)_3_	~705	0.278	0.287	0.285	0.273	0.284

**Table 5:** Intensities of absorption peaks of SNC 2.5% SiO_2_ from 0 to 120 h.

Functional Group	Wave Number (cm^−1^)	Absorbance				
		Virgin	30 h	60 h	90 h	120 h
C–H stretching in CH_3_	~2963–2940	0.109	0.109	0.111	0.111	0.112
Si–CH_3_	~1280–1260	0.473	0.471	0.473	0.481	0.485
Si–0–C	~1110–1050	0.625	0.619	0.619	0.621	0.617
Si–0–Si	~1130–1000	1.107	1.089	1.081	1.108	1.112
Si–O stretching of O–Si(CH_3_)_3_	~875–865	0.250	0.250	0.253	0.250	0.250
Si–O stretching (Si–CH_3_)_2_	~850–790	1.290	1.280	1.275	1.306	1.316
Si–C stretching of Si–(CH_3_)_3_	~705	0.277	0.277	0.280	0.279	0.278

**Table 6:** Intensities of absorption peaks of SMC 15% SiO_2_ from 0 to 120 h.

Functional Group	Wave Number (cm^−1^)	Absorbance				
		Virgin	30 h	60 h	90 h	120 h
C–H stretching in CH_3_	~2963–2940	0.113	0.109	0.110	0.109	0.111
Si–CH_3_	~1280–1260	0.464	0.465	0.461	0.472	0.479
Si–0–C	~1110–1050	0.633	0.625	0.619	0.610	0.615
Si–0–Si	~1130–1000	1.106	1.086	1.073	1.095	1.109
Si–O stretching of O–Si(CH_3_)_3_	~875–865	0.251	0.249	0.247	0.244	0.246
Si–O stretching (Si–CH_3_)_2_	~850–790	1.275	1.257	1.222	1.283	1.287
Si–C stretching of Si–(CH_3_)_3_	~705	0.281	0.281	0.282	0.280	0.282

**Table 7:** Intensities of absorption peaks of SMC 15% ATH from 0 to 120 h.

Functional Group	Wave Number (cm^−1^)	Absorbance				
		Virgin	30 h	60 h	90 h	120 h
C–H stretching in CH_3_	~2963–2940	0.094	0.111	0.098	0.106	0.109
Si–CH_3_	~1280–1260	0.360	0.417	0.366	0.417	0.433
Si–0–C	~1110–1050	0.449	0.521	0.454	0.515	0.538
Si–0–Si	~1130–1000	0.754	0.927	0.762	0.901	0.961
Si–O stretching of O–Si(CH_3_)_3_	~875–865	0.200	0.228	0.201	0.222	0.229
Si–O stretching (Si–CH_3_)_2_	~850–790	0.851	1.020	0.860	1.013	1.082
Si–C stretching of Si–(CH_3_)_3_	~705	0.269	0.308	0.273	0.292	0.295

**Table 8:** Intensities of absorption peaks of SMC 10% ATH 2% SiO_2_ from 0 to 120 h.

Functional Group	Wave Number (cm^−1^)	Absorbance				
		Virgin	30 h	60 h	90 h	120 h
C–H stretching in CH_3_	~2963–2940	0.110	0.110	0.115	0.116	0.111
Si–CH_3_	~1280–1260	0.475	0.457	0.477	0.480	0.469
Si–0–C	~1110–1050	0.606	0.600	0.595	0.599	0.606
Si–0–Si	~1130–1000	1.090	1.054	1.073	1.094	1.089
Si–O stretching of O–Si(CH_3_)_3_	~875–865	0.247	0.246	0.243	0.243	0.248
Si–O stretching (Si–CH_3_)_2_	~850–790	1.275	1.193	1.233	1.281	1.247
Si–C stretching of Si–(CH_3_)_3_	~705	0.287	0.299	0.288	0.282	0.287

## Data Availability

Author confirm the availability of all the findings in the manuscript.
